# Necrotic and apoptotic adipocytes in the hypoxic tumor microenvironment supply triglycerides to induce cisplatin resistance in the metastatic lymph nodes of head and neck carcinoma

**DOI:** 10.1038/s41419-025-08239-y

**Published:** 2025-11-24

**Authors:** Feiran Li, Huiying Huang, Qiang Huang, Xinhui Mao, Ning Cong, Wenjie Zhuang, Chi-Yao Hsueh, Ming Zhang

**Affiliations:** 1 Department of Otorhinolaryngology, Eye & ENT Hospital, Fudan University, Shanghai, China; 2 Department of Otorhinolaryngology Head & Neck Surgery, Huadong Hospital of Fudan University, Shanghai, China

**Keywords:** Cancer microenvironment, Cancer metabolism, Head and neck cancer, Metastasis, Tumour biomarkers

## Abstract

Metastatic lesions in cervical lymph nodes are generally less sensitive to induction chemotherapy than primary tumors, making cervical lymph node metastasis one of the most significant prognostic factors in head and neck squamous cell carcinoma (HNSCC). However, the underlying mechanism of cisplatin resistance in these lymph nodes remains unclear. Lipidomic analysis of 21 HNSCC patients revealed distinct lipid profiles between cervical lymph node metastases and primary tumors, with triglycerides notably enriched in the metastatic nodes, suggesting a critical role for adipocytes. Further investigation confirmed the presence of cancer-associated adipocytes within cervical lymph node metastases, which supply triglycerides to tumor cells. The hypoxic tumor microenvironment promotes apoptosis and necrosis in adipocytes, a process accelerated by hypoxic tumor cells, leading to increased triglyceride release. In HNSCC cells, triglycerides promote lipid droplet accumulation and enhance contact between lipid droplets and mitochondria via the interaction of perilipin-2 (PLIN2) and carnitine palmitoyltransferase-1A (CPT1A), thereby reversing cisplatin-induced rises in intracellular reactive oxygen species (ROS). In vivo, xenograft tumors located in adipocyte-rich regions showed larger volume and greater mass after cisplatin treatment. This study is the first to demonstrate that adipocytes are key components in cervical lymph node metastasis of HNSCC and are closely associated with cisplatin resistance. Mechanistically, the hypoxic tumor microenvironment facilitates crosstalk between tumor cells and adipocytes, increasing triglyceride supply from adipocytes. This, in turn, promotes lipid droplet–mitochondria contact in HNSCC cells through PLIN2–CPT1A binding, counteracting cisplatin-induced ROS elevation and contributing to chemoresistance

## Introduction

Head and neck squamous cell carcinoma (HNSCC) is the sixth most common malignancy, accounting for more than 800,000 new cancer cases per year and more than 7% of cancer-related deaths [1]. Cervical lymph node metastasis is one of the most important prognostic factors for HNSCC [2]. Although the treatment modality for HNSCC patients has changed from surgery-based treatment to induction chemotherapy-guided therapy, cervical lymph node metastases are often less responsive than primary tumors [3], and recurrence can occur only in the cervical lymph node metastases [4]. Our prior study also revealed that for N2/3 patients with fewer chemosensitive cervical lymph node metastases, neck dissection should be performed as soon as possible to prevent regional progression [5]. Thus, it is critical to clarify the underlying reason why cervical lymph node metastases are resistant to current cisplatin-based induction chemotherapy regimens.

As a highly heterogeneous and complex network, the tumor microenvironment comprises a variety of dynamic cells, including cancer-associated adipocytes (CAAs), which have gained increasing attention. Previous studies have shown that CAAs are associated with a poor prognosis and chemoresistance in many types of tumors [6–9]. On the one hand, adipocytes can serve as a consistent energy source for surrounding tumor cells [9]. On the other hand, they are active endocrine cells that produce a wide variety of cytokines and hormones through endocrine and paracrine mechanisms to further promote tumor progression [10, 11]. However, until now, no study has explored the potential role of these adipocytes in HNSCC or cervical lymph node metastasis.

Our previous study revealed that lymph node necrosis is a strong predictor of a poor response to induction chemotherapy in HNSCC patients [12]. When a tumor expands more than 4 mm, it is believed to be deficient in oxygen and nutrients, which may lead to tumor necrosis and treatment resistance [13, 14]. This hypoxia also affects other cell types in the tumor microenvironment, such as cancer-associated fibroblasts and immune cells [15, 16]. However, little attention has been given to the relationship between hypoxic adipocytes and tumor cells. Thus, in this study, we focused on how hypoxic adipocytes promote cisplatin resistance in the cervical lymph node metastases of HNSCC patients.

## Materials and methods

### Patients and tissue sample collection

This study was approved by the ethics committee of Eye and ENT Hospital, Fudan University (Ethical number: 2022076, ClinicalTrials.gov ID: NCT05494190). Informed consent was obtained from all subjects. Patients were pathologically diagnosed with HNSCC, and the presence of cervical lymph node metastasis was confirmed according to postoperative pathology. Patients with distant metastasis or simultaneous second primary tumors were excluded. For tissue specimen collection, patients who received preoperative treatment, including chemotherapy and radiotherapy, were also excluded. All the tissues were collected during surgery, stored at −80 °C, and processed into cryosections or paraffin sections. For patients who received induction chemotherapy, patient chemosensitivity was evaluated according to RECIST 1.1.

### Lipidomic analysis

The total lipids were extracted with chloroform-methanol (2:1) and analyzed via liquid chromatography‒mass spectrometry. Data obtained from the positive and negative scan modes were then analyzed via LipidSearch V4 to identify lipids. OPLS-DA modeling was used to assess the data quality. The preliminary screening criteria for differential lipid species were *P* > = 0.05 and VIP > = 1, and a significant difference in the abundance of lipids was defined as FC > 2, *P* < 0.001, and VIP > 1.5. The obtained data were subsequently analyzed on the BioDeep Platform (https://www.biodeep.cn).

### Tissue sections

HNSCC patients’ tumor tissues were prepared into frozen sections for Oil Red O analysis. After fixing with 4% paraformaldehyde for 15 min, sections were immersed in the Oil Red O working solution (Wuhan Guge Biological Technology, China) for 8–10 min (light-protected) and rinsed with water. Briefly differentiate sections with 75% alcohol, followed by water rinse. Then, nuclei were counterstained with hematoxylin, and the sections were mounted. The quantitative analysis of Oil Red O staining was performed using ImageJ software (National Institutes of Health, Bethesda, MD, USA).

For HE staining, paraffin sections were dewaxed and stained with hematoxylin and eosin (Shanghai Jingke Chemical Technology, China). Then, H&E-stained slides were subjected to whole-slide scanning for digital analysis. CAA were outlined according to Isnaldi et al. [17], in which CAA have been defined as the first three lines of adipocytes close to the invasive front. Normal adipocytes were outlined in the negative neck lymph nodes. The diameter and area of adipocytes were analyzed according to Palomäki et al. [18] using Qupath software (QuPath, Scotland). More than 150 adipocytes in each group were included in the final analysis.

### Cell lines and culture

3T3-L1 cells were obtained from the Cell Bank of the Shanghai Academy of Chinese Sciences. It was recently authenticated by STR testing and tested without mycoplasma contamination. Preadipocyte 3T3-L1 cells were cultured in DMEM (Gibco, USA) supplemented with 10% newborn calf serum (Every Green, China), and differentiation was induced as previously described by Furukawa et al. [19]. To prepare the adipocyte-conditioned medium, mature adipocytes were washed twice with phosphate-buffered saline and cultured in serum-free medium for 24 h. Then, the cell supernatant was collected, centrifuged at 3000 rpm for 20 min, and stored at −80 °C.

HNSCC cell lines (Fadu, TU686, and Detroit 562) were obtained from Eye and ENT Hospital, Fudan University. Fadu cells were cultured in DMEM (Gibco, USA), supplemented with 10% fetal bovine serum (BI, USA), while TU686 and D562 cells were cultured in 1640 medium (Gibco, USA), supplemented with 10% fetal bovine serum (BI, USA). They were recently authenticated by STR testing and tested without mycoplasma contamination. For hypoxia exposure, the cells were cultured in a hypoxia incubator (Thermo Scientific, USA), where the O_2_ concentration was maintained at 1%.

The coculture of adipocytes and tumor cells was conducted via a 0.4 μm pore transwell culture system (Corning, USA). Adipocytes were grown in the lower chamber, and 2 × 10^4^ tumor cells were seeded in the upper chamber to achieve indirect coculture.

siRNA was used to knockdown cellular carnitine palmitoyltransferase-1A (CPT1A) expression. siRNA sequences were obtained from RiboBio (Guangzhou, China). The CPT1A siRNA sequence for Fadu (1#) and TU686 cells (2#) were shown in the Supplementary Table 1. They were transfected with control siRNA and CPT1A siRNA using lipo8000 (Beyotime, China) according to the manufacturer’s instruction. Knockdown efficiency was assessed 48 h post-transfection by qPCR, and the result was also shown in the Supplementary Table 1.

### Drug sensitivity measurement and migration and invasion assays

The cells were seeded at 8000/well in 96-well plates overnight. The next day, after the cells had attached, the culture medium was changed, and the cells were incubated with cisplatin-containing medium according to the concentration gradient. After 48 h, a CCK8 assay kit (Topscience, China) was used to evaluate cell viability. The IC50 was calculated according to the absorbance at 450 nm.

Transwell chambers were used to assess cell migration and invasion ability. For the invasion assay, the upper transwell chamber was covered with Matrigel mixture (BD Biosciences, USA). Typically, a cell suspension prepared in serum-free medium was added to the upper chamber at a density of 1 × 10^4^/100 µL. The cells were subsequently incubated at 37 °C for 48 h. After being fixed with 4% paraformaldehyde and stained with 0.1% crystal violet, the cells on the lower surface were counted.

### Xenograft tumor model

The animal research was approved by the Animal Ethics Committee of the Eye and ENT Hospital of Fudan University. All experiments involving animals were conducted in adherence to all relevant ethical guidelines and regulations. Three- to four-week-old male BALB/c-Nude (GemPharmatech, China) were housed and raised in a specific pathogen-free environment. As this study employed a self-controlled design, randomization and blinding methods were not used. To establish xenograft models, a total of 6 × 10^6^ Fadu cells were subcutaneously injected into the left back (3 × 10^6^) and right groin (3 × 10^6^) of nude mice (5 mice). The growth of the subcutaneous tumors was monitored every 3 days, and after 25 days, cisplatin was injected intraperitoneally at 4 mg/kg BW on days 0, 7, and 14, as described by Park et al. [20]. Three days after the last cisplatin administration, the mice were sacrificed, and the tumor tissues were collected. Tumor volume and tumor mass were recorded. Tumor volume was calculated as follows: volume = (length × width^2^)/2.

### Lipid droplet staining

Both Oil Red O and BODIPY 493/503 were used to stain cellular lipid droplets in this study. The stained cells were first fixed with 4% paraformaldehyde. Oil Red O staining solution was prepared by diluting the stock solution (Oricell, China) with distilled water at a ratio of 3:2, followed by centrifugation at 250 × *g* for 4 min. Then, the cells were stained with Oil Red O at room temperature for 15 min. For BODIPY 493/503 (MCE, USA) staining, the cells in the six-well plates were incubated with 1 ml of 2 μM BODIPY 493/503 working at room temperature for 30 min.

### RNA extraction and real-time quantitative PCR

The cells were collected, and RNA was extracted using TRIzol extraction reagents. Reverse transcription was carried out via a reverse transcription kit (Accurate Biotechnology, China). qPCR was performed with SYBR Green Mix (Accurate Biotechnology, China) on a real-time PCR instrument (Applied Biosystems, USA). The primers were designed via Primer Blast. The sequences of the primers are shown in Supplementary Table 2. Relative gene expression was calculated from the obtained Ct values via the ΔΔCt method.

### Triglyceride (TG) measurement

The supernatant TG content was measured using the TG Colorimetric Assay Kit (Elabscience, China) according to the manufacturer’s recommended protocol.

### Transcriptome sequencing

Total RNA was extracted via the TRIzol method, and mRNA was purified from the total RNA via oligo-dT-attached magnetic beads. The isolated mRNA was fragmented and adaptor-ligated. These fragments were then used as templates to synthesize cDNA for library preparation. Libraries were sequenced via the Illumina sequencing platform. Differential gene expression analysis was performed via DESeq2 software, and the screening criteria were |log2FC| > = 0.585 and *P* < 0.05. These obtained data were then analyzed on the Majorbio Cloud Platform (https://cloud.majorbio.com/page/tools/). Raw data are available in a public repository (https://submit.ncbi.nlm.nih.gov/subs/sra/SUB15098266/overview).

### Apoptosis and necrosis assays

Hoechst 33342/PI staining was performed according to the manufacturer’s protocol (Beyotime, China). Then, apoptotic and necrotic cells were counted, and the percentage of apoptotic and necrotic cells was calculated.

Necrotic and apoptotic cells were then detected via Annexin V-FITC flow cytometry. Annexin V binding solution was added to the collected cells, followed by Annexin V and PI staining (Beyotime, China). The cells were analyzed with a flow cytometer (BD, USA). Separate regions of the obtained plot were used to identify cells. Annexin V+ and PI− represent early apoptotic cells, and annexin V+ and PI+ represent late apoptotic or necrotic cells.

### The preparation of TG-micelle and treatment

TG-micelle was prepared as described in Xue et al. [21]. In brief, 39 mg TG and 39 mg mPEG5000-b-PCL5000 were dissolved in the tetrahydrofuran solution (3 ml). Slowly drip the solution into ultrapure water under sonication to form an emulsion. Then, transfer the solution into a dialysis bag (molecular weight cutoff: 3500 g/mol) and dialyze against PBS for more than 24 h. The final TG-micelles were filtered through a 0.22-μm filter before use. For treatment, Fadu and TU686 cells were first starved with serum-free culture medium for 3 h, and then 0.1 mM TG was added into the culture medium.

### Reactive oxygen species (ROS) detection

The reactive oxygen species assay kit (Beyotime, China) was used for ROS detection with a DCFH-DA fluorescent probe. The DCFH-DA probe was first diluted in serum-free medium at a ratio of 1:1000 to a final concentration of 10 mM and then added to the culture plate for incubation at 37 °C for 20 min.

### Mitochondria staining

Mitochondria staining was performed by using Mitochondrial Deep Red Fluorescence Staining Kit with Mito-Tracker Deep Red FM (Beyotime, China) according to the manufacture’s protocol.

### Co-immunoprecipitation

Cells were harvested and lyzed in IP buffer containing PMSF on ice for 30 min, followed by centrifugation (10,000 rpm, 4 °C, 15 min). 0.5 mg cleared lysate was incubated with 0.5 μg Perilipin-2 (PLIN2), 0.5 μg CPT1A antibodies (or control IgG), and 20 μL pre-washed protein A/G beads overnight at 4 °C with rotation. The next day, the above was placed on the magnetic stand, washed thoroughly, and added with loading buffer. Then, boil samples with 100 μg input for 5 min. Finally, SDS-PAGE gel electrophoresis was conducted, and targeted proteins were also detected via western blot. Antibodies were shown in the Supplementary Table 3. Full and uncropped western blots were shown in the Supplementary Figs. 1–6.

### Immunofluorescent assay

Fadu and TU686 cells were used to create cell-climbing slices. After treatment, cells were fixed and permeabilized. After blocking for 30 min with BSA, the samples were first incubated with PLIN2 primary antibody at 4 °C overnight, followed by PBS washes. Subsequently, the secondary antibody was applied and incubated, then washed again. Try-594 tyramine conversion reagent was added and incubated, followed by washes. Then, stop solution was added, and the samples were incubated with the CPT1A primary antibody at 4 °C overnight. The following steps were repeated as described above. Finally, mount the slides with a DAPI-containing mounting medium. Relative antibodies were shown in the Supplementary Table 3.

### Statistical analysis

In this study, continuous data from two groups or multiple groups were compared via Student’s *t* test and one-way ANOVA, respectively. Categorical variables were analyzed via the chi-square test. All data meet the assumptions of the statistical tests used. Within each group, variability is estimated, and the variance across groups is comparable. The fluorescent or Oil Red O-positive area was quantified via ImageJ software. Statistical analysis was performed via IBM SPSS Statistics (version 23.0) and GraphPad Prism 9. We calculated the sample size for vitro and vivo experiments based on previous studies, which is commonly used in this field [6, 7]. The experiment was replicated three times. All replicates shown in the figures or used for statistical analysis referred to biological replicates, and center values were mean. The exact sample size (*n*) for each experimental group/condition was stated in the figure legend. A two-sided *P* < 0.05 was considered to indicate statistical significance. All error bars were calculated as a s.e.m.

## Results

### Cervical lymph node metastases of head and neck carcinoma are enriched with TGs

**Fig. 1:**
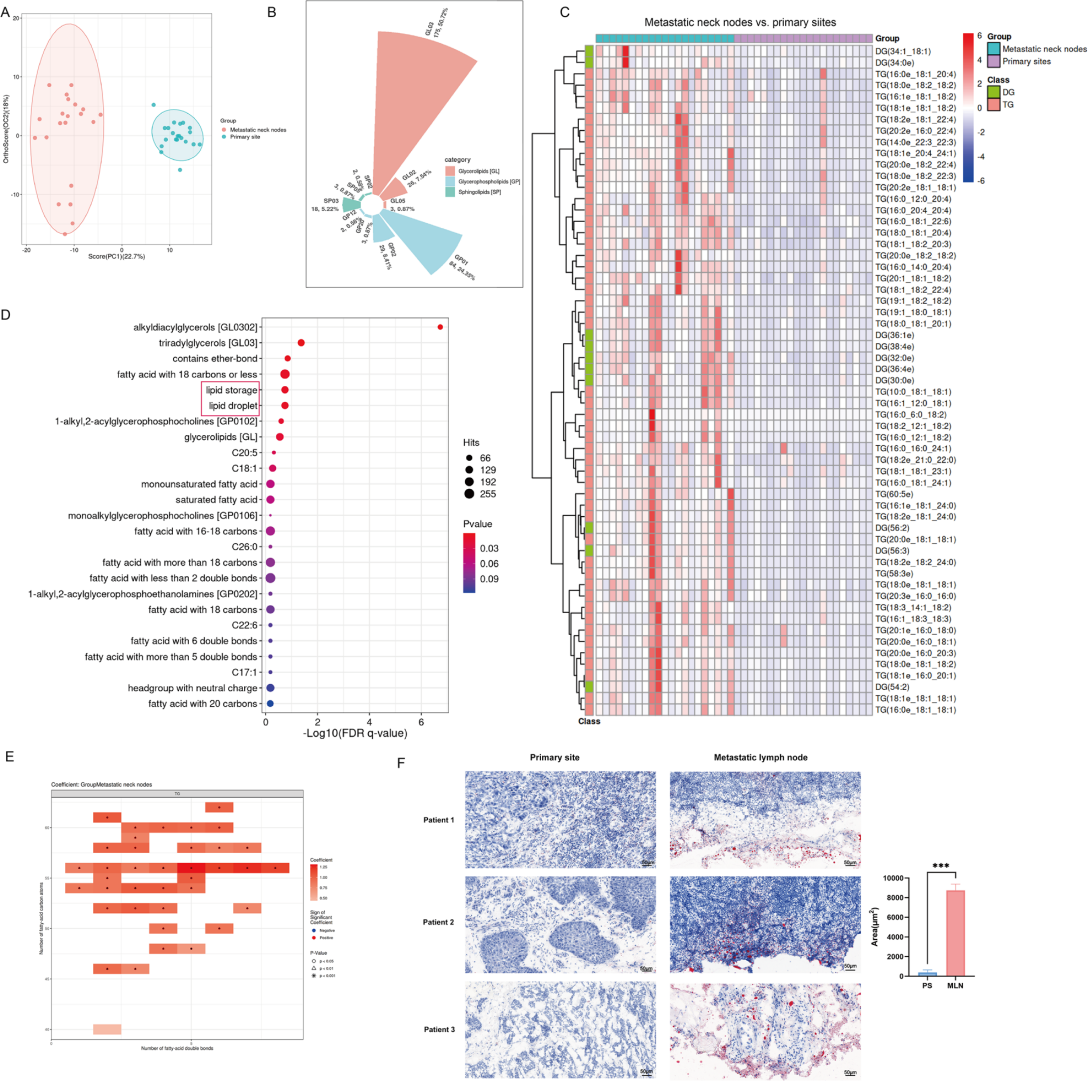
Cervical metastatic lymph nodes of head and neck carcinoma are enriched with triglyceride. **A** OPLS-DA-Score plot showed the overall differences in lipid profile of primary sites and metastatic neck nodes (*n* = 21). **B** Lipid classification of all differential lipids using lipidmaps. **C** Heatmap of differential lipids, identified by FC > 2, *P* < 0.001, VIP > 1.5. **D** Pathway enrichment analysis using the Lipid Ontology. **E** Lipid structural characteristic heatmap of triglycerides. **F** The typical images and quantitative analysis of oil red O stain area of tumor tissues from the primary sites and metastatic neck nodes in three patients. PS: primary site. MLN: metastatic lymph node. means ± SEM.*P < 0.05, ‌**P < 0.01, ‌***P < 0.001, ****P < 0.0001, ns no statistical significance.

**Fig. 2:**
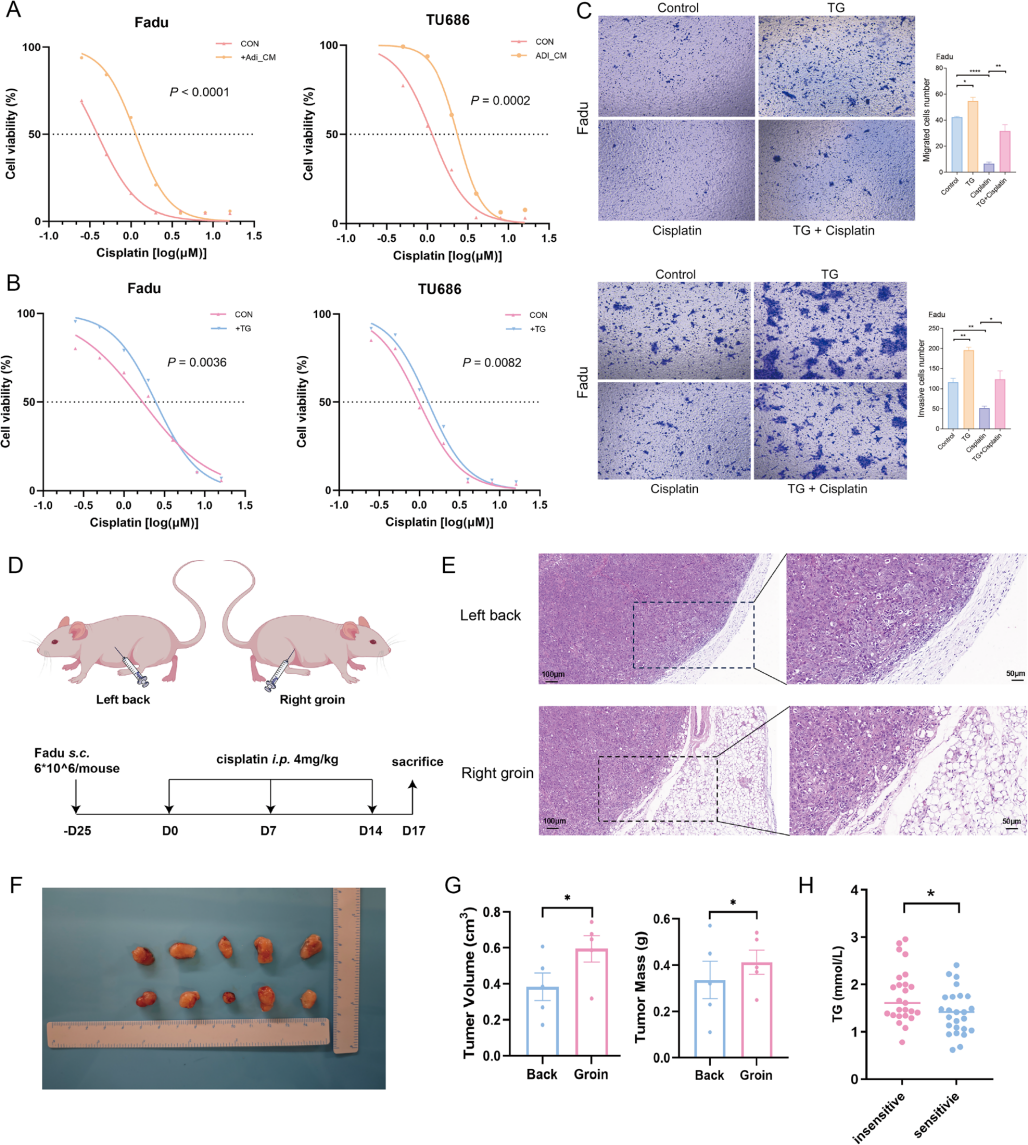
Triglyceride promotes tumor cisplatin resistance. **A** The change of cisplatin IC50 in Fadu and TU686 cells after adding adipocyte supernatant (*n* = 3). **B** The change of cisplatin IC50 in Fadu and TU686 cells after adding triglycerides (*n* = 3). **C** Migration and invasion ability changes of Fadu after adding triglycerides and cisplatin using transwell chamber (*n* = 3). **D** Schematic of BALB/c-nude mouse modeling protocol. **E** Representative HE staining pictures of left back and right groin tumors from nude mice. **F** Photos of left back and right groin tumors obtained from nude mice (*n* = 5). **G** The Volume and mass of obtained tumors were calculated (*n* = 5). **H** The serum triglycerides level of patients with sensitive (*n* = 25) and insensitive (*n* = 25) neck nodes. CM conditioned medium. TG triglyceride. means ± SEM.*P < 0.05, ‌**P < 0.01, ‌***P < 0.001, ****P < 0.0001, ns no statistical significance.

First, we collected tissue specimens from 21 HNSCC patients who underwent surgery at our hospital. The patients’ baseline characteristics are shown in Table 1. Lipidomic profile analysis of tumor tissues in the metastatic lymph nodes and primary site revealed that there was a significant difference in lipid content between the two sites, as shown in the OPLS-DA score plot (Fig. 1A). Lipid composition analysis revealed that the differential lipid species were mainly glycerolipids (Fig. 1B), and the TGs and diglycerides whose levels increased are shown in the heatmap (Fig. 2C). Lipid functional enrichment analysis revealed that the function of these elevated lipids is related to lipid droplets and lipid storage (Fig. 1D). Lipid structural analysis revealed that the levels of TGs containing long-chain polyunsaturated fatty acids increased the most in the metastatic lymph nodes (Fig. 1E). Therefore, lipidomic analysis revealed elevated levels of TGs in cervical lymph node metastases.

**Table 1:** Basic characteristics of cohort 1 patients.

	Total *n* = 21
Age	
>65	12 (57%)
Sex	
male	21 (100%)
Hypertension	
Yes	8 (38%)
Diabetes	
Yes	0 (0%)
Drinking	
Yes	18 (86%)
Smoking	
Yes	18 (86%)
Tumor site	
hypopharyngeal	15 (71%)
larynx	6 (29%)
cT classification	
1–2	11 (52%)
3–4	10 (48%)
cN classification	
1	8 (38%)
2–3	13 (62%)
C stage	
3	7 (33%)
4	14 (67%)

We further explored the location of these TGs with increased levels. Oil Red O staining of the frozen sections of the tumor tissues revealed that the TGs with elevated levels were detected mainly in adipose tissue in the tumor stroma or surrounding the tumor (Fig. 1F).

### TGs promote tumor resistance to cisplatin

Cisplatin is the main antitumor agent used in clinical practice, and we used it to evaluate the potential cancer-promoting role of TGs. First, in vitro, we assessed the impact of the adipocyte supernatant on the cisplatin IC50 in HNSCC cells and found that the adipocyte supernatant significantly increased the cisplatin IC50 value (Fig. 2A). A similar trend was observed in HNSCC tumor cells after TGs were added to the medium (Fig. 2B). Because hypopharyngeal cancer has stronger migration and invasion ability and is more prone to cervical node metastasis than other HNSCC types, we also tested the prometastatic effect of TGs on two types of hypopharyngeal cancer cells, Fadu and Detroit 562. We found that the inhibitory effects of cisplatin on migration and invasion could be reversed by TGs (Fig. 2C and Supplementary Fig. 7). These results verified that TGs facilitate HNSCC cisplatin resistance in vitro.

In an in vivo study, we evaluated the protumor effect of TGs by comparing tumors at two sites, as described by Xie et al. [22], the back (lipid-deficient tissue) and groin (lipid-rich tissue), in the same mouse after being treated with cisplatin (Fig. 2D). The HE staining results verified that the tumor tissue in the groin was surrounded by adipose tissue (Fig. 2E). Compared with those on the back, groin tumors had a greater volume and heavier mass (Fig. 2F, G). These results further verified that adipocytes and TGs can promote cisplatin resistance in HNSCC.

Next, we compared serum TG levels between chemosensitive and chemosensitive patients. The patients’ baseline characteristics were shown in Table 2. Among a total of 50 patients, the 25 patients with chemosensitive cervical lymph node metastases had significantly lower levels of serum TGs (Fig. 2H), which indicated that TGs may serve as potential biomarkers for lymph node chemosensitivity.

**Table 2:** Basic characteristics of cohort 2 patients.

	Total *n* = 50	Group 1^a^ *n* = 25	Group 2^b^ *n* = 25	*P* value
Age				0.754
>65	14 (28%)	8 (32%)	6 (24%)	
Sex				0.490
male	48 (96%)	23 (92%)	25 (100%)	
Hypertension				0.769
Yes	18 (36%)	10 (40%)	8 (32%)	
Diabetes				1.000
Yes	5 (10%)	2 (8%)	3 (12%)	
Drinking				1.000
Yes	37 (74%)	18 (72%)	19 (76%)	
Smoking				1.000
Yes	37 (74%)	18 (72%)	19 (76%)	
Subregion				0.729
Pyriform sinus	29 (58%)	14 (56%)	15 (60%)	
Postcricoid	1 (2%)	1 (4%)	0 (0%)	
Posterior pharyngeal	5 (10%)	3 (12%)	2 (8%)	
≥2 subregions	15 (30%)	7 (28%)	8 (32%)	
cT classification				0.776
1–2	28 (56%)	13 (52%)	15 (60%)	
3–4	22 (44%)	12 (48%)	10 (40%)	
cN classification				0.754
1	14 (28%)	8 (32%)	6 (24%)	
2–3	36 (72%)	17 (68%)	19 (76%)	
C stage				0.742
3	12 (24%)	7 (28%)	5 (20%)	
4	38 (76%)	18 (72%)	20 (80%)	

^a^Group 1: chemosensitive group.

^b^Group 2: chemoinsensitive group.

## CAA can provide TGs to tumor cells

**Fig. 3:**
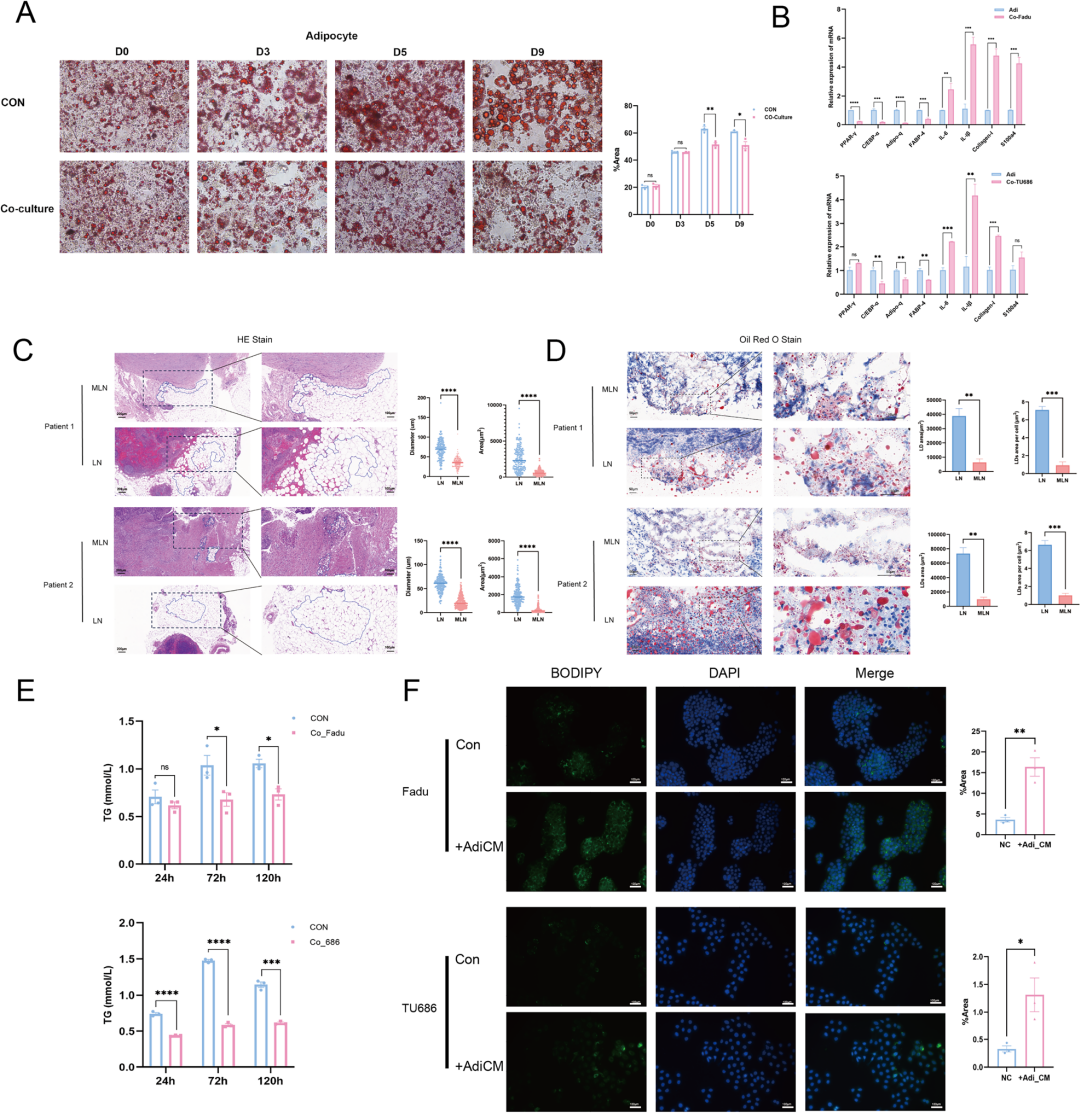
Cancer-associated adipocytes could provide triglycerides to tumor cells. **A** Typical pictures (200×) and quantitative analysis of Oil red O-stained areas in adipocytes cocultured with Fadu cells for different periods (*n* = 3). **B** The changes of relative expression level of differentiation genes, lipogenic genes, fibroblast signature genes, and cancer-promoting cell factors related genes in adipocytes after coculture with Fadu and TU686 cells (*n* = 3). **C** Typical HE staining pictures of 2 patients’ metastatic neck nodes and negative neck nodes with adipocytes circled (left). Adipocytes’ diameter and area were calculated and compared (right). **D** Typical oil red O staining pictures of 2 patients’ metastatic neck nodes and negative neck nodes with lipid droplets’ area and intensity (Oil red O staining area/cell area) calculated. **E** The level of triglycerides in the supernatant of adipocytes during coculture with fadu and TU686 cells (*n* = 3). **F** Fadu and TU686 cells’ intracellular lipid droplets were stained using BODIPY 493/503 (left), and the changes of stained area after adding adipocytes’ conditioned medium were analyzed (right, *n* = 3). Green: BODIPY. Blue: DAPI. TG triglyceride. CM conditioned medium. MLN metastatic positive neck nodes. LN negative neck nodes. LD lipid droplets. means ± SEM. *P < 0.05, ‌**P < 0.01, ‌***P < 0.001, ****P < 0.0001, ns no statistical significance.

Adipocytes around tumor cells display distinct characteristics from normal adipocytes to facilitate tumor development. To verify this phenomenon in the cervical lymph node metastases, we first cocultured mature adipocytes with Fadu cells in vitro. We found that as the co-culture time increased, the lipid droplet content in adipocytes gradually decreased, and an increasing number of large lipid droplets dispersed compared with that in adipocytes cultured alone (Fig. 3A). Additionally, these cocultured adipocytes exhibited lower levels of genes related to differentiation and adipogenesis but higher levels of fibroblast signature genes and cancer-promoting factors (Fig. 3B). These results demonstrated that cocultured adipocytes tend to dedifferentiate. Then, we outlined and compared the cell diameter and area of CAA in the sections of metastatic cervical lymph node and normal adipocytes in the sections of negative cervical lymph nodes (Fig. 3C). Also, we performed Oil Red O staining to show the distribution and size of lipid droplets (Fig. 3D). We found that, unlike normal adipocytes, adipocytes around tumor cells were smaller and had greater dispersal of intracellular lipid droplets. Thus, HNSCC cancer cells can transform normal adipocytes into CAA.

Moreover, we found that during coculture, the TG content in the supernatant of adipocytes gradually decreased (Fig. 3E), indicating that these cells may be utilized by tumor cells. To verify this hypothesis, we tested the TG content in the tumor cells through BODIPY staining. The results showed that the adipocyte supernatant significantly promoted TG accumulation in tumor cells (Fig. 3F).

## Necrotic and apoptotic adipocytes release more TGs into the tumor microenvironment

**Fig. 4:**
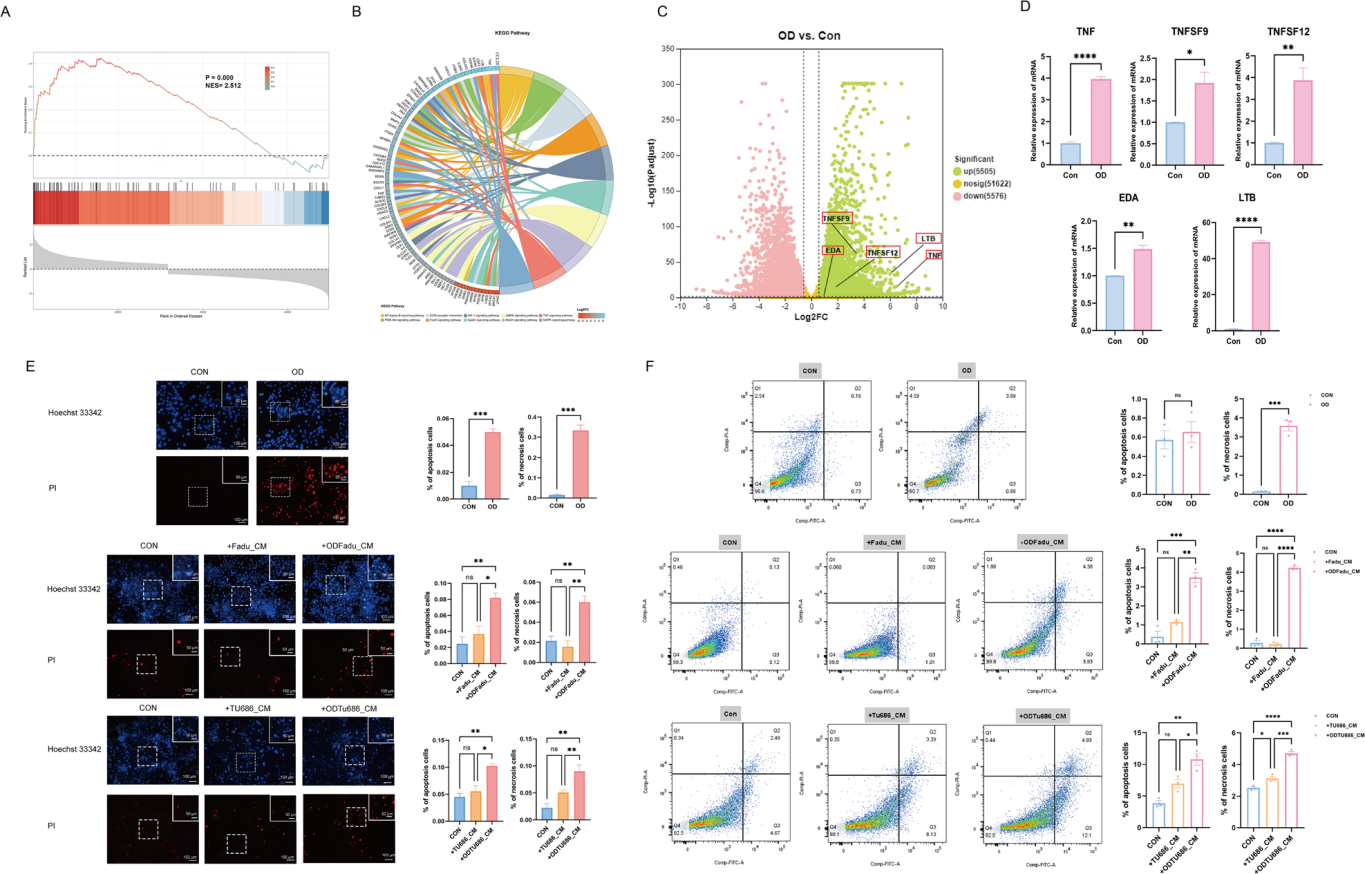
Hypoxia tumor microenvironment causes adipocytes to necrosis and apoptosis. **A**–**C** transcriptome sequencing result of hypoxia and normoxia Fudu cells. GSEA in the cytokine-cytokine receptor interaction KEGG pathway (**A**). STRING pathway enrichment relies on the KEGG database “environmental information processing” category (**B**). Volcano plots of differential genes (**C**). **D** Relative mRNA expression level of TNF superfamily. **E** Adipocyte nucleus was stained using Hoechst 33342 and PI, and the changes of apoptosis and necrosis cell rates in the hypoxia condition or after adding tumor cells’ conditioned medium were calculated. **F** Annexin/PI stain of adipocytes in the hypoxia condition or after adding tumor cells’ conditioned medium were analyzed using flow cytometry. OD oxygen deficiency. CM: conditioned medium. Blue: Hoechst 33342. Red: PI. n = 3, means ± SEM. *P < 0.05, ‌**P < 0.01, ‌***P < 0.001, ****P < 0.0001, ns no statistical significance.

Because lymph node necrosis is a common phenomenon in the cervical lymph node metastases of HNSCC patients and necrosis is often associated with hypoxia, we explored the potential influence of the hypoxic microenvironment and hypoxic tumor cells on adipocytes. The results of GSEA based on transcriptome sequencing data revealed that upregulated genes were significantly enriched in the cytokine-cytokine receptor interaction KEGG pathway (Fig. 4A) in hypoxic HNSCC cells, which indicated that hypoxic HNSCC cells actively communicate with other cells. As hypoxia is an extreme environmental condition, we explored the top 10 enriched pathways in the KEGG “environmental information processing” category. Among the top 10 enriched pathways, the TNF pathway was enriched (Fig. 4B), and 5 members of the TNF superfamily, including TNF, TNFSF9, TNFSF12, EDA, and LTB, were significantly upregulated (Fig. 4C). We then detected the expression of TNF superfamily genes via q‒PCR to further verify these findings (Fig. 4D). These results revealed that tumor cells under hypoxic conditions produce TNF-related factors, which are potent necrosis- and apoptosis-inducing factors. We then explored whether these hypoxic tumor cells could lead to adipocyte death. Preliminary nuclear staining revealed that the supernatant of hypoxic tumor cells significantly increased the proportion of apoptotic and necrotic adipocytes (Fig. 4E). Flow cytometric analysis revealed that hypoxia is a potent inducer of apoptosis and that the hypoxic tumor cell supernatant can induce both necrosis and apoptosis (Fig. 4F).

**Fig. 5:**
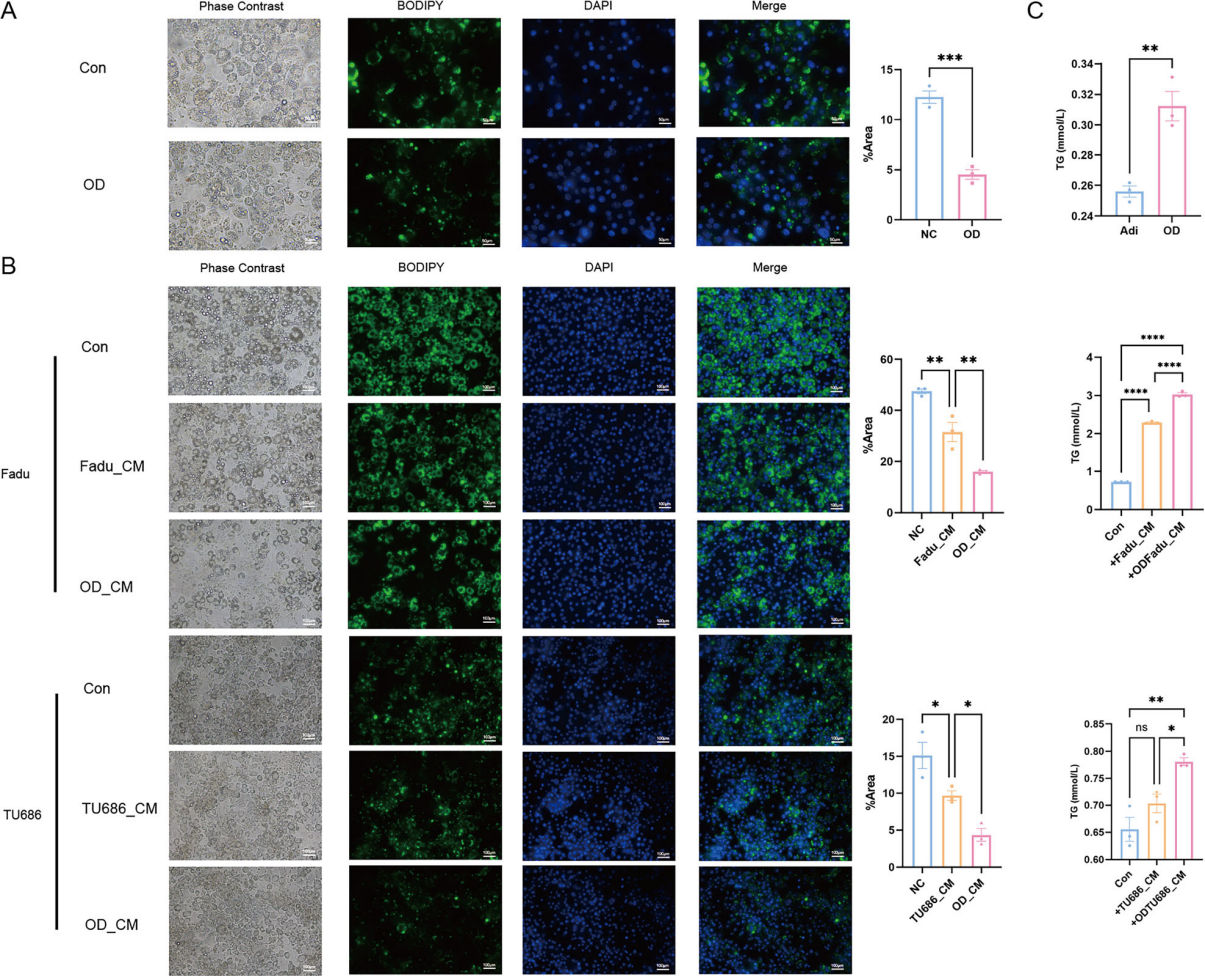
Necrosis and apoptosis adipocytes release more triglycerides to the tumor microenvironment. **A** Lipid droplets in adipocytes were stained and calculated to show the changes of intracellular triglyceride levels after exposure to hypoxia condition. **B** Lipid droplets in adipocytes were stained and calculated to show the changes of intracellular triglyceride levels after adding hypoxia or normoxia tumor cells’ conditioned medium. **C** The content of triglycerides in the adipocyte supernatant under hypoxia condition or after adding hypoxia or normoxia tumor cells’ conditioned medium. OD oxygen deficiency. CM conditioned medium. TG triglyceride. Green: BODIPY. Blue: DAPI. *n* = 3, means ± SEM. *P < 0.05, ‌**P < 0.01, ‌***P < 0.001, ****P < 0.0001, ns no statistical significance.

We then explored the impact of hypoxia and subsequent necrosis and apoptosis on the accumulation of lipid droplets in adipocytes. Compared with adipocytes under normoxic conditions, hypoxic adipocytes presented decreased intracellular lipid droplet content (Fig. 5A). Additionally, culture with the supernatant of hypoxic tumor cells led to a marked decrease in the content of adipocyte intracellular lipid droplets (Fig. 5B). Moreover, we detected the TG content in the adipocyte supernatant and found that the TG content significantly increased after exposure to hypoxia or hypoxic tumor cell supernatants (Fig. 5C). Thus, the hypoxic microenvironment can lead to adipocyte death and the release of intracellular TGs into the microenvironment, and this effect can be further strengthened by hypoxic tumor cells and exert a tumor-protective effect.

## TGs reversed the cisplatin-induced HNSCC cell death and ROS elevation in HNSCC cells

**Fig. 6:**
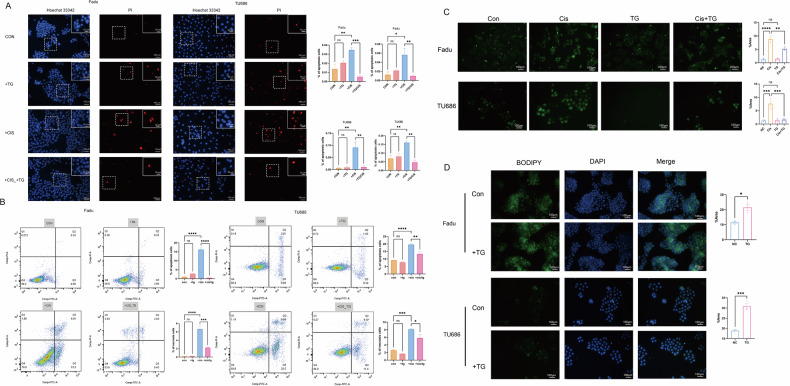
Triglyceride could reverse cisplatin-induced cell death and ROS elevation in the head and neck carcinoma cells. **A** Fadu and TU686 cell nuclei were stained using Hoechst 33342 and PI, and the changes of apoptosis and necrosis cell rates after adding triglycerides or/and cisplatin were calculated. **B** Annexin/PI stain of Fadu and TU686 cells after adding triglycerides or/and cisplatin were analyzed using flow cytometry. **C** ROS stain using DCFH-DA fluorescent probe in Fadu and TU686 cells after adding triglycerides or/and cisplatin. **D** Lipid droplet stain using BODIPY 493/503 in Fadu and TU686 cells with their area calculated after adding triglycerides. TG triglyceride. Cis cisplatin. Green: ROS (**C**), BODIPY (**D**). Blue: Hoechst 33342(**A**), DAPI (**D**). Red: PI. *n* = 3, means ± SEM. * *P* < 0.05, ‌** *P* < 0.01, ‌*** *P* < 0.001, **** *P* < 0.0001, ns no statistical significance.

To investigate the anti-cisplatin effect of TGs, we first assessed if TGs could reverse cisplatin-induced apoptosis and necrosis in HNSCC cells. The results of Hoechst/PI staining and flow cytometry showed that cisplatin significantly increased the percentage of apoptotic and necrotic cells, which could be reversed by TGs. (Fig. 6A, B). Then, we investigated if this effect was associated with ROS. Our results showed that the cisplatin-induced increase in ROS level could also be reversed by adding exogenous TGs to tumor cells (Fig. 6C). As lipid droplets are vital organelles for redox regulation, we investigated the changes in the intracellular lipid droplet content in HNSCC cells after the addition of TGs to the supernatant. The results showed that TGs significantly increased the lipid droplet content in Fadu and TU686 cells (Fig. 6D). Thus, TGs can protect HNSCC cells from cisplatin-induced ROS accumulation by increasing the intracellular lipid droplet content.

## TG promotes the binding of lipid droplets and mitochondria in HNSCC cells through PLIN2 and CPT1A

**Fig. 7:**
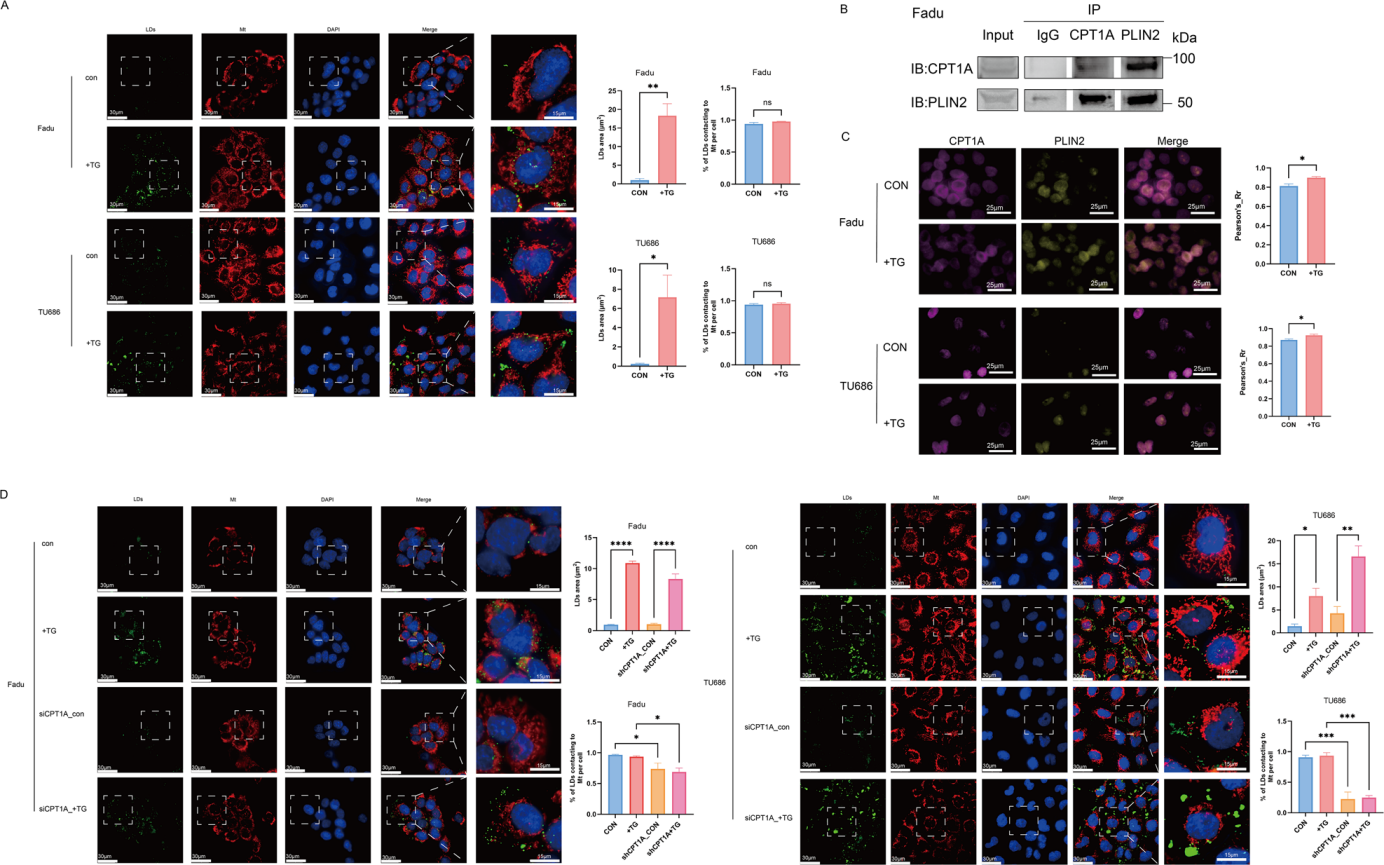
Triglyceride promotes the binding of lipid droplets and mitochondria in head and neck carcinoma cells through PLIN2 and CPT1A. **A** Intracellular lipid droplets and mitochondria of Fadu and TU686 cells were stained (Left). The area of lipid droplets and the percentage of lipid droplets contacting mitochondria after adding triglyceride were calculated and analyzed (right, *n* = 3). **B** Co-immunoprecipitation experiment to detect the binding of PLIN2 and CPT1A. **C** Immunofluorescence imaging was used to analyze the colocalization between PLIN2 and CPT1A proteins in Fadu and TU686 cells (*n* = 3). **D** CPT1A knockdown reduced the binding of lipid droplets and mitochondria in Fadu and TU686 cells (*n* = 3). TG triglyceride. LD lipid droplet. Mt mitochondria. HNSCC head and neck squamous cell carcinoma. Green: lipid droplets. Red: mitochondria. Blue: DAPI. means ± SEM. *P < 0.05, ‌**P < 0.01, ‌***P < 0.001, ****P < 0.0001, ns no statistical significance.

Our further study explored by which mechanism TGs can protect HNSCC cells from cisplatin-induced ROS accumulation. As mitochondria is known as the primary sites of ROS generation and lipid droplets could play a mitochondria-protecting role, we then stained intracellular lipid droplets and mitochondria. The results showed that TGs promoted the accumulation of intracellular lipid droplets, which increased the contacting of lipid droplets and mitochondria in Fadu and TU686 cells (Fig. 7A). Then, we explored the critical proteins that participated in this binding. As the binding of PLIN2 in the lipid droplets and CPT1A in the mitochondria has been recently discovered in the liver carcinoma [23], we test if this binding also exists in the HNSCC cells. We performed the co-immunoprecipitation experiment and immunofluorescence assay and found that PLIN2 could bind to CPT1A in HNSCC cells. Furthermore, TGs could improve this binding (Fig. 7B, C). Lastly, we knockdown CPT1A in Fadu and TU686 cells and found that accumulated lipid droplets induced by adding TGs could not bind to mitochondria (Fig. 7D). Thus, triglycerides could promote the binding of lipid droplets and mitochondria through PLIN2 and CPT1A to reverse cisplatin-induced ROS elevation.

## Discussion

**Fig. 8:**
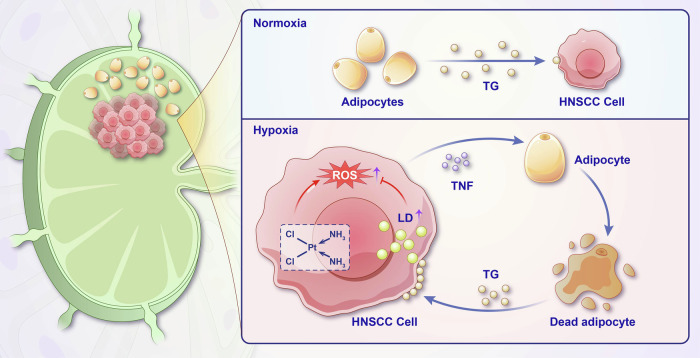
Schematic diagram of this study. The interactions of adipocytes and head and neck carcinoma cells in the metastatic neck nodes are explored. Adipocytes supply triglycerides to tumor cells, and this role could be strengthened in the hypoxia tumor microenvironment due to the death of adipocytes.

To our knowledge, our study is the first to explore the potential role of adipocytes in metastatic lymph nodes. We found that adipocytes provide TGs to tumor cells. This effect was strengthened under hypoxic conditions, which may explain the different regional responses after induction chemotherapy (Fig. 8). Previous studies have shown that adipocytes are a significant component of the metastatic bone marrow [24]. It has been reported that adipocytes and the omega-6 polyunsaturated fatty acid arachidonic acid can attract cancer cells to the metabolically active red marrow of bone [25]. Additionally, Epstein–Barr virus can induce adipocyte dedifferentiation to provide a protumorigenic microenvironment in the bone marrow of nasopharyngeal cancer patients [26]. A previous study also discovered that TGs could be released by adipocytes in the form of vesicles [27], and they could be absorbed in tumor cells through micropinocytosis [21]. Although we did not observe that hypoxic HNSCC tumor cells could absorb adipocyte-released fatty acids in our study, our results still support the conclusion that adipocytes are an important energy source in the tumor metastatic microenvironment.

As energy sources, adipocytes were also closely associated with tumor chemoresistance in previous studies [11, 28]. Our study revealed that the supernatant of adipocytes could significantly decrease the sensitivity of HNSCC cells to cisplatin, which indicates that the lipid-rich environment in cervical lymph node metastases may be a critical cause of the chemoresistance of HNSCC cells. Mechanistically, we found that TGs released from adipocytes could also reverse the cisplatin-induced increase in intracellular ROS. These results are in accordance with those of several studies showing that adipose-derived exosomes suppress ferroptosis, which is characterized by ROS accumulation, thus promoting oxaliplatin resistance in colorectal cancer [11]. However, it has also been reported that ovarian cancer cells cocultured with adipocytes express high levels of CD36 and that the inhibition of CD36 attenuated adipocyte-induced lipid droplet accumulation and reduced the ROS content [29]. Therefore, the relationship between the number of intracellular lipid droplets and the ROS content still needs to be clarified.

Lipid droplets and mitochondria are closely linked in cellular metabolism. Mitochondria are known as the primary site for ROS generation. Also, mitochondria are vulnerable to ROS assault, leading to elevated ROS generation and establishing a vicious cycle [30]. Previous studies have found that lipid droplets could serve as a protective barrier for mitochondria [31]. The physical contact between LDs and mitochondria sequesters free fatty acids, preventing the accumulation of cytotoxic lipid species and shielding mitochondria from ROS-induced damage. This mechanism promotes mitochondrial homeostasis and thereby exerts cytoprotective effects [32]. Recently, more and more studies have focused on the potential proteins that participated in this conjunction [33]. It is reported that phosphofructokinase, liver type (PFKL), promotes the binding of PLIN2 to CPT1A, which is essential for liver tumor growth [23]. These findings are in line with our results that TG-induced intracellular lipid droplets accumulation could increase their binding with mitochondria through PLIN2 and CPT1A, which could protect mitochondria from ROS attack and intracellular ROS elevation.

In clinical practice, detecting lymph node necrosis in HNSCC patients is not rare. Multiple clinical studies in HNSCC have demonstrated that necrosis is an independent factor of treatment resistance. The results of a long-term study in 757 HNSCC patients revealed that, compared with that of patients without necrotic tumors, the complete response rate of patients with necrotic tumors after treatment was significantly decreased [34]. Additionally, a study in 498 HNSCC patients revealed that tumor necrosis significantly decreased patient sensitivity to induction chemotherapy [35]. It is believed that hypoxia is one critical reason for tumor necrosis. Hypoxia is an important feature of the tumor microenvironment. Based on previous findings, our study focused not only on hypoxic tumor cells but also on the impact of hypoxia on adipocytes and subsequent tumor chemoresistance effects. Several studies have shown that hypoxic adipocytes can contribute to tumor progression through derived exosomes, metabolic reprogramming, or the modification of gene expression [36–38]. Our study revealed that hypoxia-induced death of adipocytes promoted the release of TGs to fuel tumor cells. Researchers have also reported the value of dead cells in the tumor microenvironment. The presence of necrotic cells can enhance angiogenesis and the proliferation of endothelial cells [14]. Necrotic cells also produce a specific series of endogenous molecules named damage-associated molecular patterns that can stimulate various chemoresistance-related signaling pathways in targeted cells [13]. Thus, further study on necrotic adipocytes in the tumor microenvironment and targeting strategies may provide more ideas for overcoming tumor chemoresistance.

Patients’ blood lipid profiles could provide critical and direct clues for diagnosis and treatment. Prior studies have shown that the blood lipid profile of patients with tumors is related to prognosis. One clinical study in patients with breast cancer revealed that for patients receiving neoadjuvant chemotherapy, dyslipidemia, including increased blood TG levels, is associated with a poor prognosis [39]. Additionally, in multiple myeloma, researchers have reported that the blood lipid profile is a critical prognostic factor in patients [40]. Our study revealed that TG metabolism is involved in tumor chemoresistance and that blood TG levels may be potential predictors of the regional response to induction chemotherapy in the future. However, owing to its relatively small sample size and the retrospective nature of this study, further verification studies are needed.

## Conclusion

As an important component of the cervical lymph node metastases of HNSCC, adipocytes play an important role in promoting tumor cisplatin resistance. The hypoxic tumor microenvironment promotes crosstalk between tumor cells and adipocytes by increasing the triglycerides supply of adipocytes. This promotes the contacting of lipid droplets and mitochondria in HNSCC cells through the binding of PLIN2 and CPT1A and reverses cisplatin-induced ROS elevation, which preliminarily explains why cervical lymph node metastases are prone to cisplatin resistance and provides a potential target for future therapy.

## Supplementary information

Supplementary Table 1

Supplementary Table 2

Supplementary Table 3

Supplementary Figure 1

Supplementary Figure 2

Supplementary Figure 3

Supplementary Figure 4

Supplementary Figure 5

Supplementary Figure 6

Supplementary Figure 7

## Data Availability

The datasets generated by RNA sequences in the current study are available in the NCBI repository, https://submit.ncbi.nlm.nih.gov/subs/sra/SUB15098266/overview. Other datasets generated and/or analyzed during the current study are available from the corresponding author on reasonable request.
