# Supplementary material for: Necrotic and apoptotic adipocytes in the hypoxic tumor microenvironment supply triglycerides to induce cisplatin resistance in the metastatic lymph nodes of head and neck carcinoma
Source: Cell Death Dis. 2025 Nov 24;16(1):854. doi: 10.1038/s41419-025-08239-y (PMC12644729; doi:10.1038/s41419-025-08239-y)

| CPT1A（human）ID:1374-siRNA-227(1#) | CCAUGAAGCUCUUAGACAATT |
| --- | --- |
|  | UUGUCUAAGAGCUUCAUGGTT |
| CPT1A（human）ID:1374-siRNA-1774(2#) | UGGCAAACGACGUGGAUUUTT |
|  | AAAUCCACGUCGUUUGCCATT |


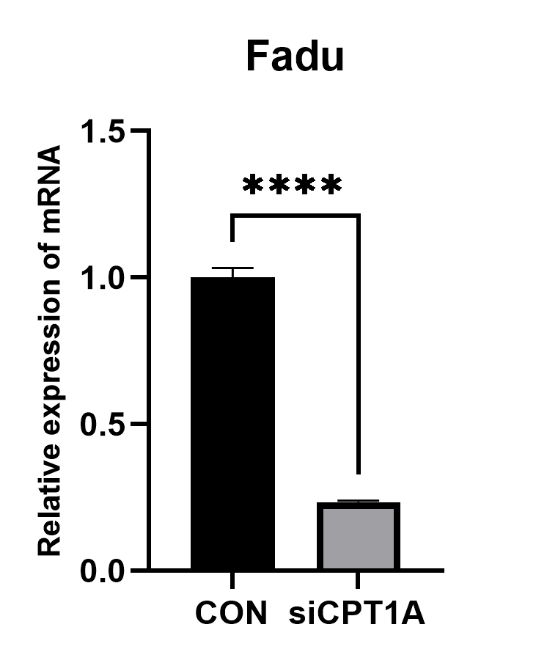

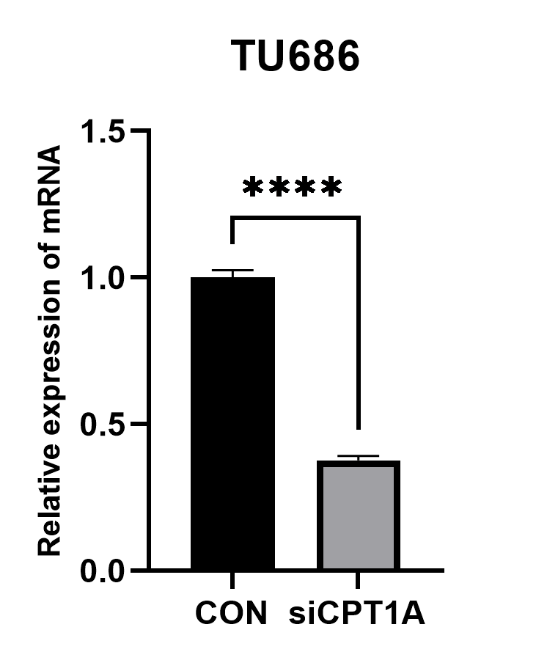

Supplement: Supplementary file 1 — Supplementary Table 1 [file 41419_2025_8239_MOESM1_ESM.docx]
