# Supplementary material for: Necrotic and apoptotic adipocytes in the hypoxic tumor microenvironment supply triglycerides to induce cisplatin resistance in the metastatic lymph nodes of head and neck carcinoma
Source: Cell Death Dis. 2025 Nov 24;16(1):854. doi: 10.1038/s41419-025-08239-y (PMC12644729; doi:10.1038/s41419-025-08239-y)
Supplement: Supplementary file 2 — Supplementary Table 2 [file 41419_2025_8239_MOESM2_ESM.docx]

Mouse

| Gene name | Sequence |
| --- | --- |
| β-actin2-F | ACTGTCGAGTCGCGTCCA |
| β-actin2-R | TCATCCATGGCGAACTGGTG |
| PPAR-γ_F | TTGCTGTGGGGATGTCTCAC |
| PPAR-γ_R | AACAGCTTCTCCTTCTCGGC |
| C/EBP-α_F | CGGTGGACAAGAACAGCAAC |
| C/EBP-α_R | ACGTTGCGTTGTTTGGCTTT |
| Adipoq_F | ATCTGGAGGTGGGAGACCAA |
| Adipoq_R | GGGCTATGGGTAGTTGCAGT |
| FABP4_F | TCACCATCCGGTCAGAGAGT |
| FABP4_R | CTGTCGTCTGCGGTGATTTC |
| IL-6_F | CTTCTTGGGACTGATGCTGGT |
| IL-6_R | GTTGGGAGTGGTATCCTCTGTG |
| IL-1β_F | AATCTCGCAGCAGCACATCA |
| IL-1β_R | GAAGGTCCACGGGAAAGACA |
| collagen I_F | AGCACGTCTGGTTTGGAGAG |
| collagen I_R | GACATTAGGCGCAGGAAGGT |
| S100a4_F | CAGGCAAAGAGGGTGACAAGT |
| S100a4_R | GCTGTCCAAGTTGCTCATCAC |

Human

| Gene name | Sequence |
| --- | --- |
| GAPDH_F | TCCAAAATCAAGTGGGGCGA |
| GAPDH_R | AAATGAGCCCCAGCCTTCTC |
| TNF_F | CTGGGCAGGTCTACTTTGGG |
| TNF_R | GAGCCAGAAGAGGTTGAGGG |
| TNFSF9_F | GGGCCTGAGCTACAAAGAGG |
| TNFSF9_R | AAGTGAAACGGAGCCTGAGC |
| TNFSF12_F | TGGGAGGAAGCCAGAATCAA |
| TNFSF12_R | CAGCAAGTCCAGCTTCAGGTA |
| LTB_F | AGCCACTTCTCTGGTGACCT |
| LTB_R | GCCGTCTCCGTTACCAGTC |
| EDA_F | TGGGTTTCTTTGGCCTCTCG |
| EDA_R | TCCGAGCGCAACTCTAGGTA |
