# Supplementary material for: Necrotic and apoptotic adipocytes in the hypoxic tumor microenvironment supply triglycerides to induce cisplatin resistance in the metastatic lymph nodes of head and neck carcinoma
Source: Cell Death Dis. 2025 Nov 24;16(1):854. doi: 10.1038/s41419-025-08239-y (PMC12644729; doi:10.1038/s41419-025-08239-y)
Supplement: Supplementary file 3 — Supplementary Table 3 [file 41419_2025_8239_MOESM3_ESM.docx]

| Antibodies | Company | Catalog No. | Dilution ratio |
| --- | --- | --- | --- |
| CPT1A Polyclonal antibody | Proteintech, China | #15184-1-AP | 0.5μg for 0.5mg total protein lysate in co-immunoprecipitation; 1:5000 for western blot;  1:1000 for immunofluorescence |
| Perilipin-2 (E6G6M) Rabbit mAb | Cell Signaling Technology, USA | #95109 | 0.5μg for 0.5mg total protein lysate in co-immunoprecipitation;  1:200 for immunofluorescence |
| Perilipin-2 Monoclonal antibody | Proteintech, China | # 60340-1-Ig | 1:500 for western blot |
| Anti-mouse secondary antibody | Abmart, China | #M21001 | 1:5000 |
| Anti-rabbit secondary antibody | Abcam, USA | # Ab6721 | 1:1000 |
