# Supplementary figures and images for: Necrotic and apoptotic adipocytes in the hypoxic tumor microenvironment supply triglycerides to induce cisplatin resistance in the metastatic lymph nodes of head and neck carcinoma
Source: Cell Death Dis. 2025 Nov 24;16(1):854. doi: 10.1038/s41419-025-08239-y (PMC12644729; doi:10.1038/s41419-025-08239-y)

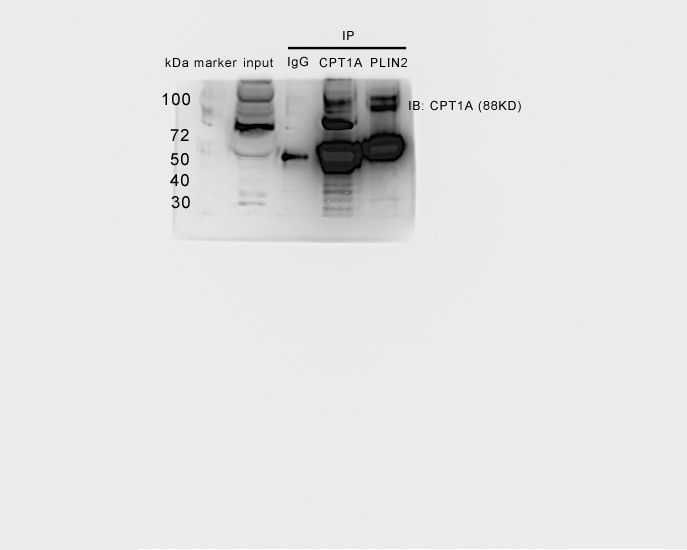

Supplement: Supplementary file 4 — Supplementary Figure 1 [file 41419_2025_8239_MOESM4_ESM.tif]

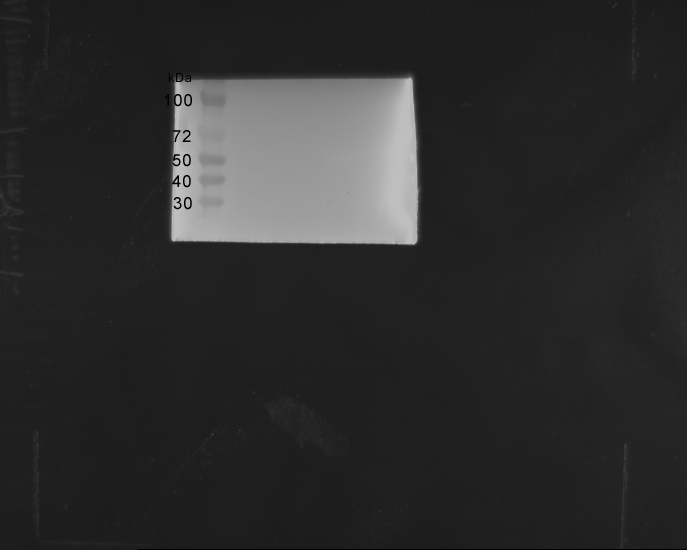

Supplement: Supplementary file 5 — Supplementary Figure 2 [file 41419_2025_8239_MOESM5_ESM.tif]

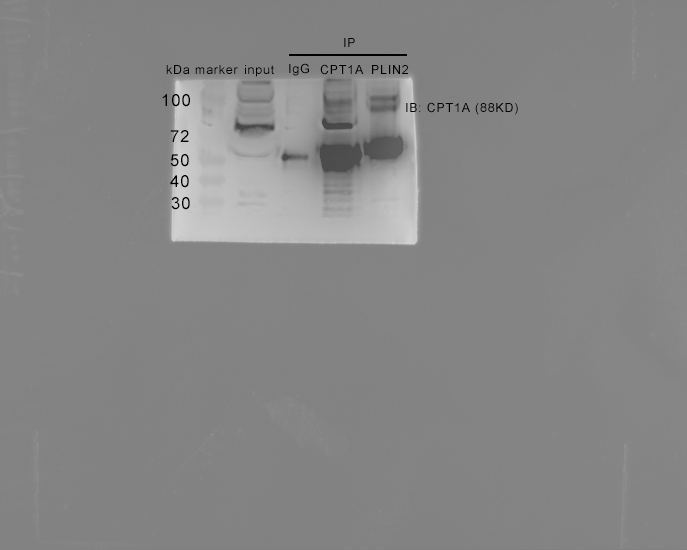

Supplement: Supplementary file 6 — Supplementary Figure 3 [file 41419_2025_8239_MOESM6_ESM.tif]

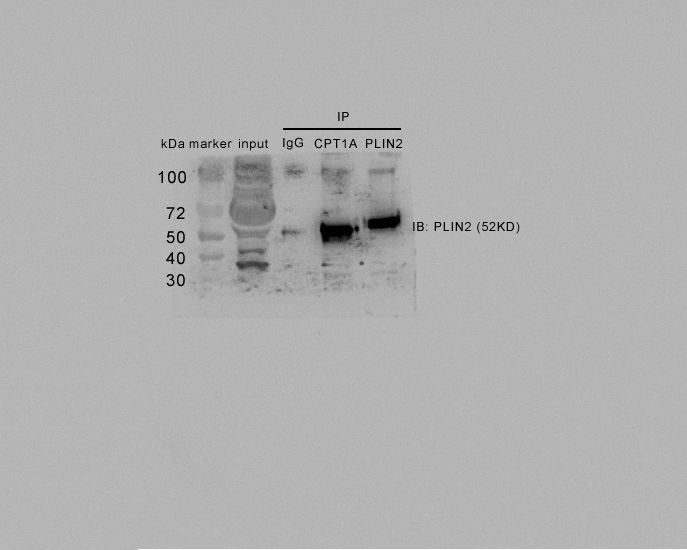

Supplement: Supplementary file 7 — Supplementary Figure 4 [file 41419_2025_8239_MOESM7_ESM.tif]

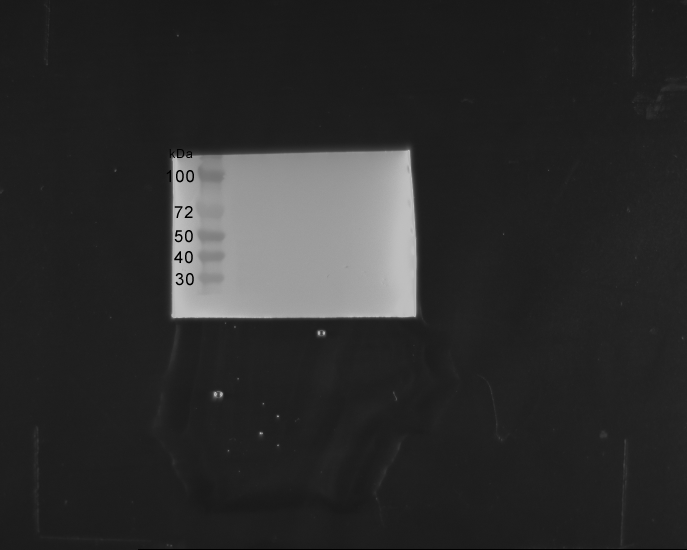

Supplement: Supplementary file 8 — Supplementary Figure 5 [file 41419_2025_8239_MOESM8_ESM.tif]

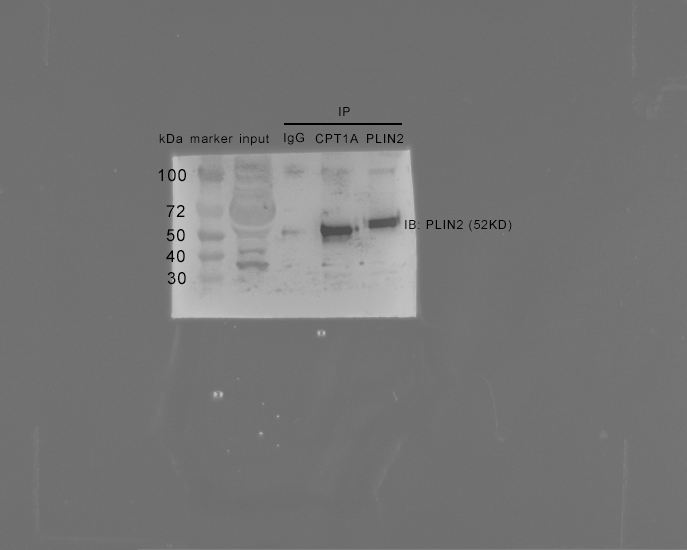

Supplement: Supplementary file 9 — Supplementary Figure 6 [file 41419_2025_8239_MOESM9_ESM.tif]

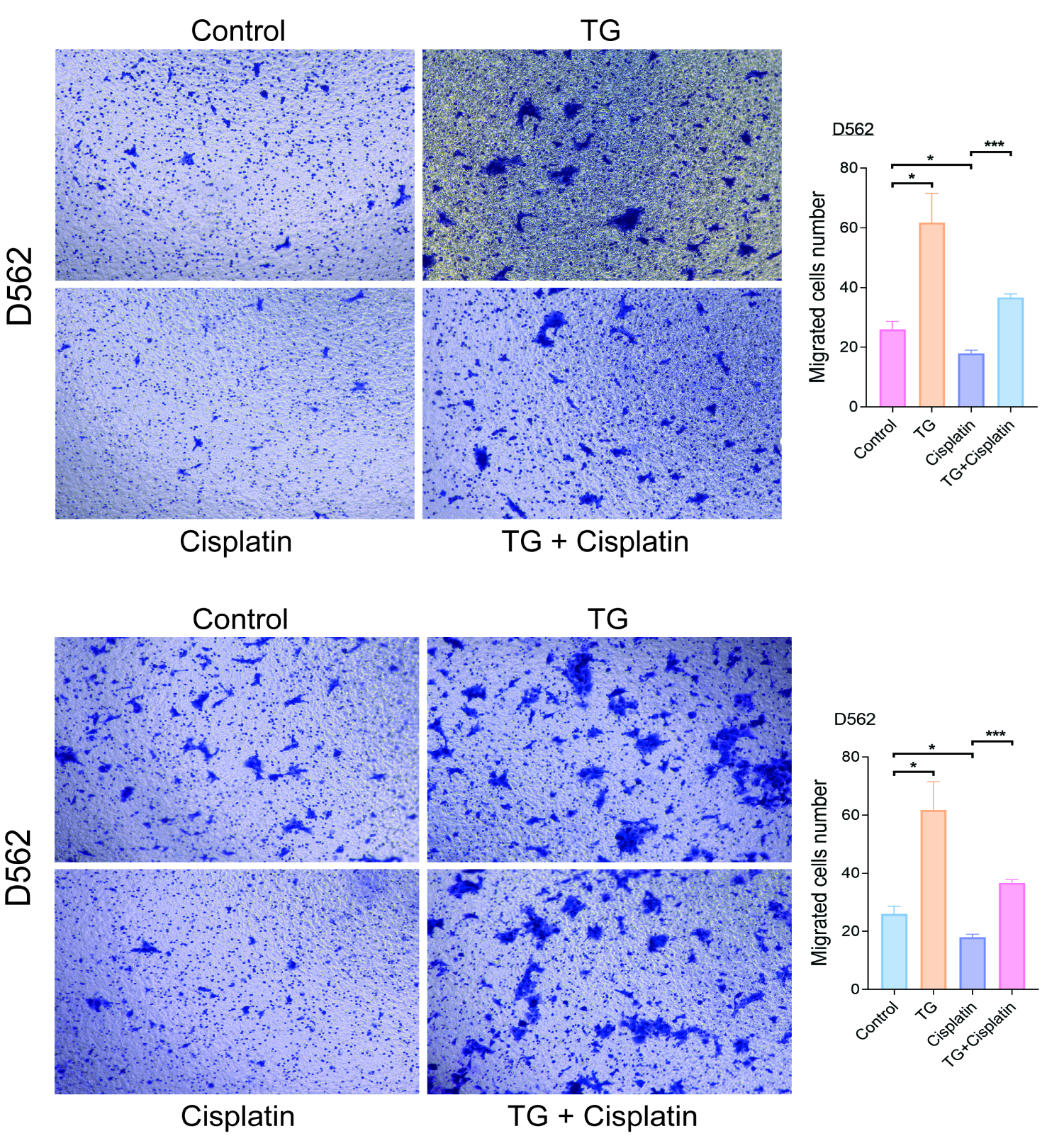

Supplement: Supplementary file 10 — Supplementary Figure 7 [file 41419_2025_8239_MOESM10_ESM.tif]
